# Bilateral Septic Arthritis of the Knees in a Patient With Hair-Dye Poisoning

**DOI:** 10.7759/cureus.43853

**Published:** 2023-08-21

**Authors:** Bhargav K M, Virali Gulla, Ragamayi K, Susmith Koneru, Jayaprada Rangineni

**Affiliations:** 1 Medicine, Sri Venkateswara Institute of Medical Sciences, Tirupati, IND; 2 Orthopaedics, Balaji Institute of Surgery, Research and Rehabilitation for the Disabled (BIRRD), TIrupati, IND; 3 Microbiology, Sri Venkateswara Institute of Medical Sciences, Tirupati, IND

**Keywords:** supervasmol, steroid use, bilateral knee swelling, hair dye poisoning, septic arthritis

## Abstract

Bilateral septic arthritis (SA) is a rare presentation that is often associated with sepsis syndrome in patients with underlying risk factors. We present the case of a 25-year-old female with laryngeal oedema due to para phenylene diamine-containing hair-dye poisoning. She was receiving treatment with intravenous (IV) methylprednisolone and IV antibiotics along with airway management. The hospital course was complicated by new acute onset of bilateral knee pain and swelling while being on antibiotics. Arthrocentesis revealed purulent synovial fluid, which on culture grew *Staphylococcus haemolyticus*. The patient underwent arthroscopic washouts with IV and intra-articular antibiotic treatment. IV corticosteroid treatment could be the possible risk factor for systemic bacteraemia in this patient.

## Introduction

The ingestion of hair dye for self-poisoning with a suicidal intention is found to be increasing in developing countries of Africa and Asia, especially India [1,2]. Super Vasmol 33 (Hygienic Research Institute Pvt. Ltd, Mumbai, India) is a paraphenylenediamine (PPD)-containing, emulsion-based hair dye that is commonly used for coloring hair in India [3].

We report the rare and unusual occurrence of bilateral septic arthritis (SA) of the knee joints in a patient with Super Vasmol 33 poisoning. SA of the knee is an emergency requiring prompt diagnosis and treatment. It is usually monoarticular, with an incidence of 2-3/1,00,000 [4]. Polyarticular SA (PASA) accounts for 15% of all infectious arthritides with *Staphylococcus aureus* being the most commonly isolated organism [4]. Immunosuppression due to corticosteroid administration for the Super Vasmol 33 poisoning could have been a risk factor in the present case.

## Case presentation

A 25-year-old female presented with an alleged history of consumption of about 200mL of Super Vasmol about 72 hours prior to admission. She complained of difficulty in breathing and swallowing; cervicofacial oedema was evident on physical examination. She was intubated on the day of admission in view of impending respiratory distress. On examination, the patient was afebrile with a blood pressure of 100/70 mmHg, heart rate of 100 beats/minute, and respirations of 22 cycles/minute. She received treatment with IV antibiotics (Inj. ceftriaxone 1 gm IV twice daily) for 14 days, IV methylprednisolone 500 mg once a day for three days, and other supportive treatment including chest physiotherapy. The laboratory investigations at the time of admission (Table 1) showed elevated levels of serum creatine kinase (CK) (CK-myocardial band (MB) of 218 IU/L) and myoglobinuria, suggestive of rhabdomyolysis.

**Table 1:** Laboratory investigations CK-MB: creatine kinase-myocardial band; INR: international normalised ratio; TSH: thyroid stimulating hormone

Observation	Date				Unit	Reference range
	October 23, 2021	October 29, 2021	November 3, 2021	November 14, 2021		
Haemoglobin	13.8	12.8	15.3	9.8	g/dL	12-16
Total leucocyte count	15800	23500	23300	13700	cells/cumm	4000-11000
Platelet count	3,10,000	3,82,000	2,33,000	7,32,000	Lakh/cumm	1.5-4.0
C- reactive protein	25.36	195.78	135.8	28.12	mg/L	<6.00
Serum cortisol			31.4		µg/dL	6.70-22.60
Procalcitonin	0.19	13.94	41.87	7.82	ng/ml	0
Prothrombin time		14.7		17.2	Seconds	11-16
INR		1.26		1.47		
Serum calcium	8.1			8.2	mg/dL	8-10.50
Serum CK-MB			218		IU/L	5-25
Serum creatinine	0.43	0.46	0.5	0.37	mg/dL	0.30-1.30
Serum phosphorus				3.8	mg/dL	2.50-4.80
Serum magnesium				2	mg/dL	1,50-3
Serum potassium	4.4	4.7	4.8	5.4	mmol/L	3.50-5
Serum sodium	134	139	132	136	mmol/L	135-145
Serum urea	51	46	32	22	mg/dL	10-40
TSH			2		mIU/L	0.50-5
Urine for haemoglobin	Negative			Negative	Descriptive	Negative
Urine for myoglobin	Positive			Negative	Descriptive	Negative

On day 5 of admission, she developed tachycardia. Electrocardiogram (ECG) showed incomplete left bundle branch block; serum troponin I was elevated (6.42 ng/mL). In view of persistent oropharyngeal oedema and slow weaning from the ventilator, one more course of IV methylprednisolone was given. A tracheostomy was done on day 14 of admission as she had ventilator-associated pneumonia.

**Figure 1:**
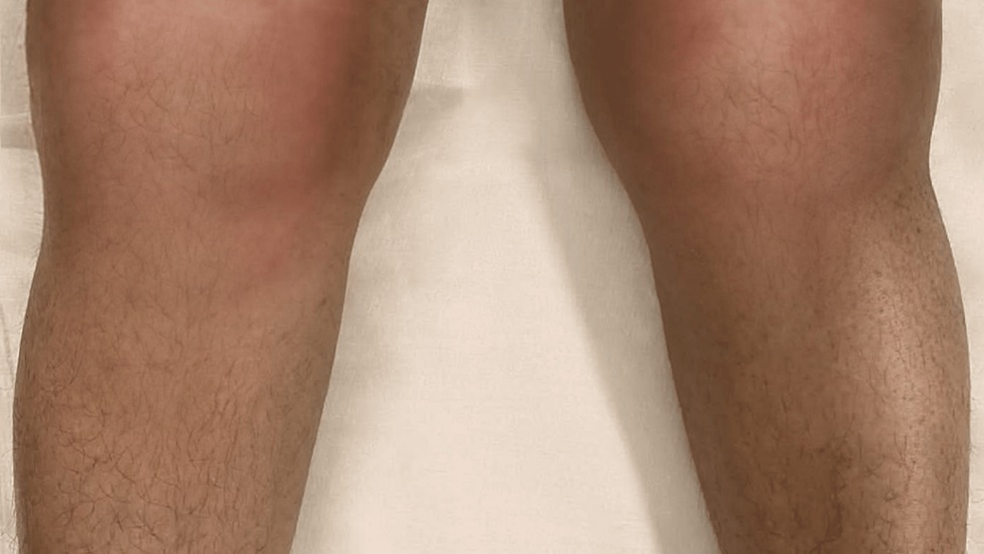
Bilateral knee swelling and erythema

Her clinical condition was gradually improving. On day 21 of admission, seven days after completion of two weeks of ceftriaxone, she developed swelling and pain in both knee joints (Figure 1) along with a high fever. On examination, movements were restricted at both the knee joints, and gross swelling, raised temperature, and tenderness were noted. Ultrasonography of the knees showed right knee joint effusion with internal echoes, thickened synovium and increased vascularity and left knee joint effusion.

**Figure 2:**
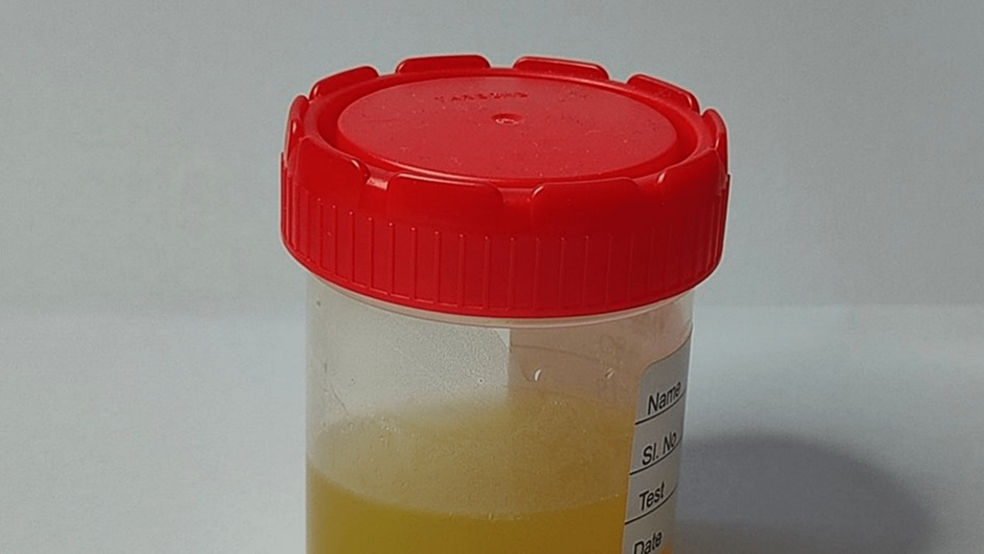
Right knee joint aspirate

Arthrocentesis yielded 35 mL frank pus (Figure 2) and the culture of pus and blood grew *Staphylococcus haemolyticus* (sensitive to cefoxitin, ciprofloxacin, clindamycin, co-trimoxazole, gentamicin, linezolid, vancomycin, and tigecycline and resistant to ampicillin, erythromycin, and penicillin). Synovial fluid analysis results are shown in Table 2. Antibiotics were changed to IV Injection linezolid 600 mg twice daily and IV injection cefepime-tazobactam 1.125 gm twice daily as per the culture and sensitivity reports and the above antibiotics were continued for a period of two weeks. Repeated arthroscopic washouts with vigorous antibiotic coverage for a total period of eight weeks resulted in clinical improvement with no residual functional disability.

**Table 2:** Left knee joint synovial fluid analysis AFB: acid-fast bacillus

Synovial fluid analysis	Result	Unit	Reference range
Color and transparency	Greenish and turbid	Descriptive	Colorless and transparent
Total leukocyte count	64,000	Cells/cumm	Less than 200
Neutrophils	90	%	Inflammatory>50; Septic>90
Lymphocytes	10	%	Around 15%
AFB smear	No AFB seen	Descriptive	No AFB seen
Gram stain	Moderate number of pus cells, no organisms seen	Descriptive	
Culture	Staphylococcus haemolyticus	Descriptive	Sterile

The patient recovered and was discharged in stable condition from the hospital on day 30 of admission. She continued to receive arthroscopic washouts for six weeks with antibiotic coverage. She continues to do well on follow-up. Her clinical condition improved and was advised physiotherapy for an additional period of four weeks.

## Discussion

This is a unique case of bilateral SA in a patient following poisoning with Super Vasmol 33 hair dye. Prolonged use of corticosteroids and bacteraemia during treatment could be the possible risk factors for the occurrence of this rare association.

Deliberate poisoning with Super Vasmol 33, an emulsion-based hair dye, is on a rising trend due to its easy availability and low cost. The constituents of this dye are PPD, resorcinol, propylene glycol, ethylenediaminetetraacetic acid (EDTA) sodium [3]. PPD causes cervicofacial oedema and rhabdomyolysis leading to acute tubular necrosis and renal failure. It can also cause direct cardiotoxicity leading to fatal arrhythmias [5]. Propylene glycol has been implicated in causing high anion gap metabolic acidosis, hyperosmolarity, and arrhythmias [2]. EDTA sodium can lead to hypocalcemia.

 In a study based on clinical profile and outcomes of hair dye poisoning, it was found that 88% of them had cervicofacial oedema, with positive outcomes after the use of hydrocortisone and methylprednisolone [6].

SA is an acute inflammation of synovial membranes with purulent effusion into joints as a result of bacterial infection secondary to hematogenous spread, direct inoculation, or contiguous spread from an adjacent source [7]. The annual incidence of SA ranges from two to three per 1,00,000 population [4]. The risk factors for the occurrence of SA include age (>60 years), recent bacteraemia, degenerative joint disease, rheumatoid arthritis, and corticosteroid therapy. In addition to the above, patients with diabetes mellitus, leukaemia, cirrhosis, cancer, intravenous drug abuse, and on cytotoxic chemotherapy have a higher incidence of SA [4].

Bilateral SA is a rare condition, most commonly due to *Staphylococcus aureus *followed by *Streptococcus pyogenes* and *Streptococcus pneumonia* [7]. This is a unique case with the occurrence of bilateral SA due to *Staphylococcus haemolyticus* bacteraemia. There are very limited studies on the occurrence of bilateral SA. One such study investigated the occurrence of bilateral SA due to group B *Streptococcus* infection. It was observed in this study that streptococcal SA generally occurs in older women with the possibility of diabetes mellitus being a predisposing factor [8]. There are several published reports of the occurrence of pneumococcal SA. One such report highlights the rare occurrence of bilateral pneumococcal SA in a patient with systemic bacteraemia [9]. This case also shed light on the involvement of other joints involving shoulder and wrist joints in addition to knee joints [10]. One such interesting case is the occurrence of bilateral simultaneous pneumococcal SA in a patient with Felty’s syndrome following splenectomy [11].

The risks and benefits associated with prolonged use of steroids, along with the possibility of occurrence of rare presentations of bilateral SA need to be evaluated further. Prednisolone of more than 10 mg/day or a cumulative dose of more than 700 mg poses a risk of infection [12].

Although a number of case reports have been published on the occurrence of bilateral SA, we believe that this is one of the first cases of simultaneous bilateral SA in a case of Super Vasmol 33 hair dye poisoning following steroid use occurring due to *Staphylococcus hemolyticus* infection. This case illustrates the importance of immediate evaluation for SA in any patient with systemic bacteraemia and bilateral swollen joints. Bilateral swollen and painful knees can occur due to bilateral SA, rather than as a flare-up of osteoarthritis or reactive arthritis. In a patient suspected to have bilateral SA, underlying risk factors for its occurrence need to be identified. Vigorous treatment with arthroscopic washouts and antibiotic therapy helps prevent mortality.

## Conclusions

We believe that this is one of the first cases reports of simultaneous bilateral SA in a case of Super Vasmol 33 hair dye poisoning following steroid use, occurring due to *Staphylococcus hemolyticus* infection. Distinguishing SA from other conditions, identifying underlying risks, and aggressive management through arthroscopic washouts and targeted antibiotics are vital to prevent mortality. 
